# Undulatory Propulsion at Milliscale on Water Surface

**DOI:** 10.1002/advs.202309807

**Published:** 2024-03-14

**Authors:** Ziyu Ren, Kagan Ucak, Yingbo Yan, Metin Sitti

**Affiliations:** ^1^ School of Mechanical Engineering and Automation Beihang University Beijing 100191 China; ^2^ Physical Intelligence Department Max Planck Institute for Intelligent Systems 70569 Stuttgart Germany; ^3^ Laboratory for Multiscale Mechanics and Medical Science SV LAB School of Aerospace Xi'an Jiaotong University Xi'an 710049 China; ^4^ Institute for Biomedical Engineering ETH Zurich Zurich 8092 Switzerland; ^5^ School of Medicine and College of Engineering Koç University Istanbul 34450 Turkey

**Keywords:** bioinspiration, miniature robotics, soft robotics, undulatory propulsion, water surface

## Abstract

The oscillatory pitch motion at the leading edge of a millimeter‐scale flexible sheet on the water surface can generate undulatory locomotion for swimming, similar to a honeybee vibrating its wings for propulsion. The influence of various parameters on such swimming strategy remains unexplored. This study uses magnetic milliswimmers to probe the propulsion mechanics and impact of different parameters. It is found that this undulatory propulsion is driven by capillary forces and added mass effects related to undulatory waves of the milliswimmers, along with radiation stress stemming from capillary waves at the interface. Modifying the parameters such as actuation frequency, pitch amplitude, bending stiffness, and hydrofoil length alters the body waveform, thus, affecting the propulsion speed and energy efficiency. Although undulatory motion is not a prerequisite for water surface propulsion, optimizing body stiffness to achieve a proper undulatory waveform is crucial for efficient swimming, balancing energy consumption, and speed. The study also reveals that the induced water flow is confined near the water surface, and the flow structures evolve with varying factors. These discoveries advance the understanding of undulatory water surface propulsion and have implications for the optimal design of small‐scale swimming soft robots in the future.

## Graphical abstract

The water‐surface propulsion of an undulatory swimmer stems from the complex interplay of various forces that are generated by the body's waveform and the waves at the water‐air interface. Adjusting the material and dimensions of the body, along with the excitation manner of the head, allows for tuning of the swimmer's waveform that is crucial for optimizing propulsion performance.

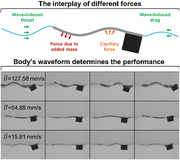

## 1 Introduction

The graceful movement of creatures on water surface has long captivated scientists and spurred innovation. This dynamic interface, regulated by hydrodynamic forces, such as fluid drag, buoyancy, and surface tension, has led to the evolution of diverse and intricate locomotion strategies in various organisms. From the agile strides of lizards^[^ ^1^ ^]^ to the graceful glide of water striders;^[^ ^2^ ^]^ from the snails that employ pedal waves for crawling underneath the water surface^[^ ^3^ ^]^ to the insects harnessing Marangoni flows through surfactant emission for propulsion,^[^ ^4^ ^]^ nature's repertoire of movement strategies on the water surface is vast and mesmerizing.

Engineers have been inspired by animal locomotion on water surfaces to create new robots. Basilisk lizard‐inspired legged robots utilize the impact force stemming from high stepping speeds between their feet and the water surface to dash across it.^[^ ^5^ ^]^ Water strider‐inspired robots harness the curvature force and momentum transfer during their leg rowing strokes to glide gracefully and energy efficiently on water.^[^ ^2^, ^6^ ^]^ Robots without movable parts have been designed to generate a surface tension gradient that triggers Marangoni flows for propulsion.^[^ ^7^ ^]^ Mimicking the beetle larvae, a magnetically actuated sheet‐shaped soft millirobot can climb upward along the water meniscus and land on the ground by deforming the air‐water interface.^[^ ^8^ ^]^ Furthermore, an insect‐scale jumping robot mimicking the jumping mechanism of fleas achieves higher take‐off velocities on water surfaces than on land by adopting a bioinspired curved leg tip design.^[^ ^9^ ^]^

Intriguingly, when trapped in water, honeybees propel themselves by flapping their wetted wings.^[^ ^10^ ^]^ This vibration‐induced locomotion is particularly compelling to engineers, as it obviates the need for complex transmission systems and intricate structural designs, which is particularly advantageous for small‐scale robots with limited onboard space and weight.^[^ ^11^ ^]^ Such vibrational propulsion has also been adapted for locomotion on solid terrains.^[^ ^11^, ^12^ ^]^ When subjected to vibrations on the water surface, a momentum flux, driven by surface waves, can develop. A fore‐aft asymmetric wave creates an imbalanced momentum flux, resulting in net thrust. This principle underpins the propulsion of a robot with a high‐stiffness body that exhibits small deformation during actuation.^[^ ^11c^ ^]^ In contrast, honeybees generate wave‐like motions on their flexible wings using leading‐edge actuation on the water surface,^[^ ^10^ ^]^ which is potentially instrumental in propulsion. The impact of such body flexibility and its wave‐like motion in propulsion on the water surface is not well understood yet.

This study delves into the complexities of undulatory propulsion on the water surface at the milliscale utilizing magnetically actuated milliswimmers. By inducing a pitch motion at their heads, we generate a wave that propagates through their passive flexible bodies. Using these milliswimmers as experimental platforms, we systematically examine the influence of various parameters on propulsion performance. These parameters include actuation frequency, pitch amplitude, body bending stiffness, and body length. We extract body waveform profiles from video recordings to assess kinematic quantities and compute hydrodynamic forces, assuming a 2D motion. We also employ flow visualization techniques, including the Schlieren imaging and particle image velocimetry, to offer insights into the water surface topology and the flow velocity field surrounding the swimmers.

## 2 Results

### 2.1 Achieving Undulatory Propulsion on Water Surface

**Figure 1:**
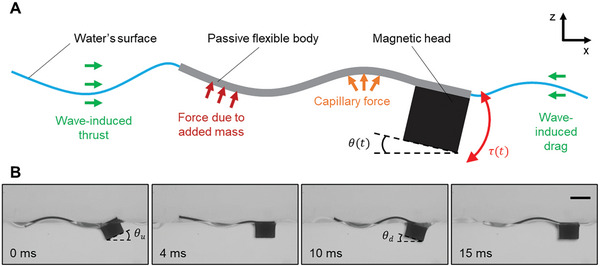
Undulatory swimming on the water surface (at the air‐water interface). A) A periodic pitch motion is generated by the oscillating magnetic torque on the magnetic head of the swimmer. Such motion at the leading edge of the flexible body induces the undulatory motion of the flexible body. B) Body waveforms of the milliswimmer from the side‐view video snapshots. The pitch angle amplitude θ_p_ = θ_u_  + θ_d_. Scale bar is 2 mm.

The milliswimmer consists of a magnetic head and a flexible tail as depicted in **Figure** **1**A. When the head is subjected to an oscillating magnetic field with a sinusoidal waveform in the *z*‐direction, it undergoes a periodic pitch motion due to the induced oscillating magnetic torque. This periodic pitch motion generates a wave transmitting along the flexible body (Figure 1B), which is constructed from a polymer elastomeric thin film. This wave arises from the synergy between the body's elastic bending and the hydrodynamic forces acting upon it. The head's oscillation frequency matches that of the external magnetic field. Nevertheless, the relation between the magnetic field and the pitch angle amplitude (θ_p_ = θ_u_  + θ_d_) is intricate, due to the considerable influence of hydrodynamic forces. To achieve a specific head pitch angle amplitude, it is imperative to calibrate the magnetic field for each frequency and body design.

### 2.2 Forces Influencing the Swimmer Propulsion

**Figure 2:**
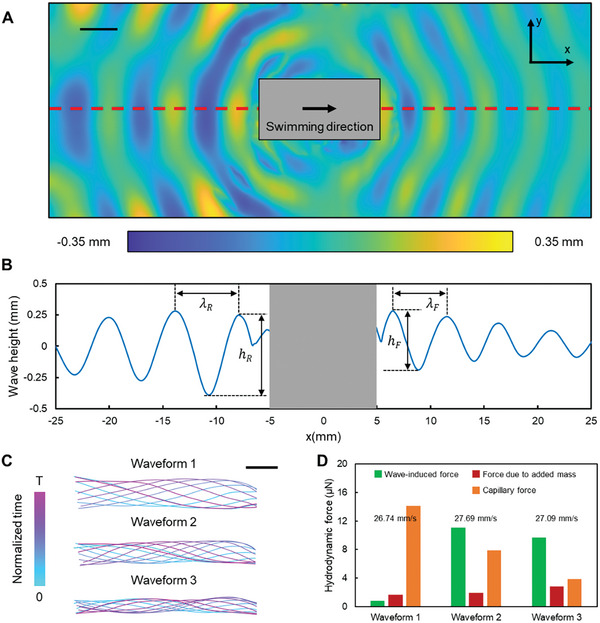
Forces contribute to the undulatory propulsion on the water surface. A) The surface waves are reconstructed using the Schlieren imaging technique. The scale bar is 5 mm. B) The wave profile is extracted from the red dashed line in (A). C) The tail deformation profiles of three swimmers with similar swimming speeds but different body bending stiffness. The scale bar is 2 mm. D) Different forces are induced by three waveforms. The average swimming speed of each swimmer is indicated in the plot. The forces.

Previous studies have employed the calculation of radiation stress by reconstructing the surface morphology to estimate the thrust of propulsors.^[^ ^10^, ^11^ ^]^ The waves generated by the swimmer's body are observable using the Schlieren technique (**Figure** **2**A, Experimental Section: Surface wave reconstruction, Movie S1, Supporting Information). The wave amplitude along the body's central axis, denoted by the red dashed line in Figure 2A, is illustrated in Figure 2B. Both amplitude and wavelength notably vary between the front and rear waves. The amplitude of the rear wave is larger than the front wave. The swimmer's forward motion causes the rear wave to stretch and the front wave to compress due to the Doppler effect, leading to λ_R_ > λ_F_. The values of λ_R_ and λ_F_ are less than 10 mm, suggesting the waves at the water surface are capillary waves.

**Figure 3:**
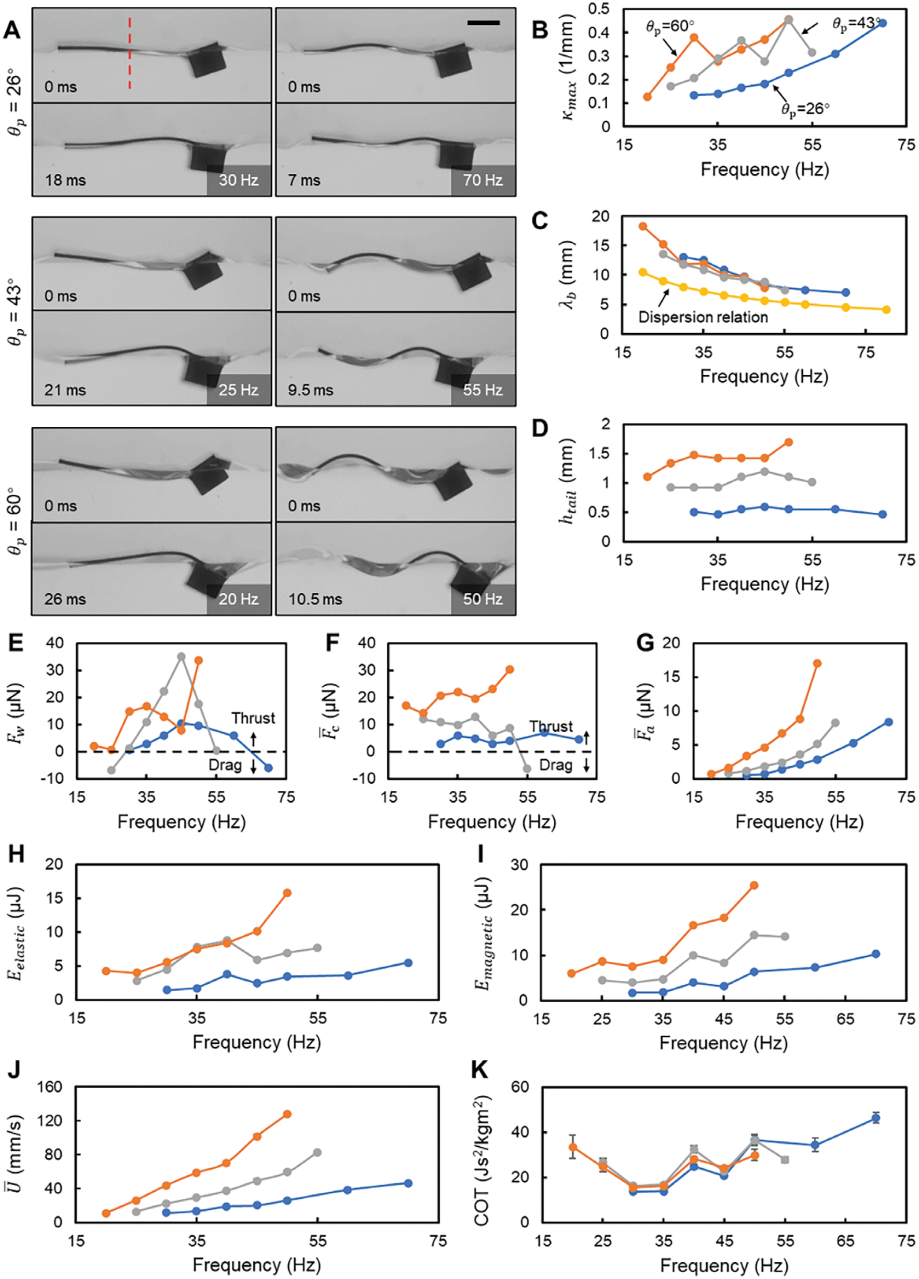
The influence of varying the pitch angle amplitude, θ_p_. A) The video snapshots of the swimmers with different θ_p_ from the side view. The red dashed line indicates the location to measure κ_max_. Scale bar is 2 mm. B–D) Body waveform parameters, including the maximum curvature at the middle part of the body, κ_max_, the average wavelength of the body waveform, λ_b_, and the peak‐to‐peak amplitude of the tail tip, *h*_tail_. E–G) Forces acting on the swimmer during propulsion, including the wave‐induced force, *F*_w_, the time‐averaged capillary forces along *x* direction, $\bar{F_{c}}$, and the time‐averaged forces due to added mass, $\bar{F_{a}}$. H,I) The maximum elastic energy due to the body's bending, *E*_elastic_, and the work done by magnetic torque in one cycle, *E*_magnetic_. J,K) The average swimming speed, $\bar{U}$, and cost of transport, *COT*. In (B–I), the value of each data point is calculated from the body profile extracted from one period. In (J,K), the value of each data point is averaged from five independent trials, and the error bar represents the standard deviation. The error bars on some points are invisible due to the small standard deviation.

Three different swimmers were constructed, each with distinct body bending stiffness. By adjusting the magnetic field magnitude and frequency, they were able to swim at approximately the same speed, while their body waveforms differed noticeably (Figure 2C). With a 2D motion assumption, the thrust forces stemming from the surface waves were estimated^[^ ^11c^ ^]^ (**Figure** **3**D, Experimental Section: Estimation of the wave‐induced force). It's worth noting that, despite the swimmers moving at comparable speeds, their wave‐induced thrusts (*F*_w_) are not consistent (0.82, 11.08, and 9.72 µN). This observation indicates that the surface wave profile alone is insufficient for determining the quality of the propulsion, underscoring the necessity of considering the interaction between the body's waveform and the surrounding fluid to comprehensively assess the swimmer's propulsion performance. In view of this, we need to incorporate other forces that may act as thrust or drag.

We assume that the propulsion is markedly affected by the body's waveform, predominantly through two primary forces. The first originates from the rapid flapping of the body. The milliswimmers operate within a frequency range of 20–80 Hz. Such high‐frequency actuation causes the robot body to flap against the water surface rapidly. This fast movement alternately accelerates and decelerates the fluid below, increasing the undulating body's effective mass and triggering an inertial force on the body. This “added mass” force, which had been previously used to calculate the force needed to quickly lift a circular disk from the water surface,^[^ ^13^ ^]^ is utilized to determine the force due to fluid acceleration. The second force stems from the interface deformation. The swimmer's body causes deformation in the water's surface, resulting in a pressure difference across its flexible body. This pressure difference can produce a capillary force perpendicular to the local interface, playing a crucial role in propulsion. Using a 2D model, the *x* component of the forces due to added mass (*F*_a_, Experimental Section: Estimation of the force due to added mass) and the deformed interface (*F*_c_, Experimental Section: Estimation of the capillary force) can be computed from the side‐view body kinematics.

Across these swimmers, the average forces per actuation period, $\bar{F_{a}}$ and $\bar{F_{c}}$, are comparable in magnitude to *F*_w_, indicating the critical role each force plays in propulsion (Figure 2D). Summing up *F*_w_, $\bar{F_{a}}$, and $\bar{F_{c}}$ can estimate the total thrusts for waveforms 1–3, which are 16.62, 20.89, and 16.45 µN, respectively. In waveform 1, $\bar{F_{c}}$ counterbalance the small *F*_w_ and $\bar{F_{a}}$. In waveforms 2 and 3, the elevated value of *F*_w_ compensates the small $\bar{F_{a}}$ and $\bar{F_{c}}$. It should be noted that when the swimmer's body becomes extremely stiff, the undulatory body waves disappear, making the magnitudes of *F*_a_ and *F*_c_ several orders smaller than *F*_w_ (Figure S1, Supporting Information). This underscores the body's waveform is pivotal to the generation of *F*_a_ and *F*_c_.

Subsequent sections will delve into the systematic variations of frequency (*f*), head pitch amplitude (θ_p_), body bending stiffness (*EI*), and body length (*L*). These factors are explored to understand their influence on the body waveform and, consequently, on propulsion performance.

### 2.3 Influence of the Head Pitch Angle

As the first parameter, we varied θ_p_ (26°, 43°, and 60°) while fixing *L* = 10 mm and *EI* = 1.83 × 10^−10^ Nm^2^. The side‐view body kinematics at different *f* and θ_p_ values can be seen in Figure 3A and Movie S2 (Supporting Information). By extracting body profiles from the videos, we quantified the body kinematics by measuring the body's midpoint curvature (marked by the red dashed line in Figure 3A), the average wavelength, and the tail tip amplitude (Experimental Section: Body profile extraction and waveform parameter calculation).

The maximum curvature (κ_max_) at the body's midpoint provides insights into the body's bending state. A rise in *f* increases κ_max_, indicating more significant bending at higher frequencies (Figure 3B). A similar trend is observed with an increase in θ_p_. We also compared the average body's wavelength (λ_b_) with the capillary wave's wavelength (λ_d_) derived from the dispersion relation with a deep‐water assumption.^[^ ^14^ ^]^ The body waveform is synergistically governed by the hydrodynamic forces and the bending stiffness of the flexible sheet. This comparison aims to evaluate the dominance of the body's bending stiffness and the hydrodynamic forces on the formation of the body's waveform. As *f* increases, the difference between λ_b_ and λ_d_ narrows, indicating growing influence from hydrodynamic forces with a higher frequency (Figure 3C). The tail tip amplitude (*h*_tail_) positively correlates with the thrust generated by the swimmer's rear wave. The results indicate that *h*_tail_ increases with θ_p_ (Figure 3D). However, *h*_tail_ does not vary drastically with *f*. In fact, except for θ_p_ = 60°, *h*_tail_ of the other cases all have a decreasing tendency at high *f*.

Because *F*_w_ incorporates the influence of both the front and rear waves, *F*_w_ may also turn negative and act as a drag (Figure 3E). Generally speaking, a larger *h*_tail_ can induce a larger *F*_w_ although a few exceptional points exist. The curved body induces non‐negligible $\bar{F_{c}}$, which may act as thrust or drag (Figure 3F). The highest value of $\bar{F_{c}}$ appears at θ_p_ = 60° due to its pronounced curvature. Across all trials, forces due to the added mass provide a consistent thrust source for the undulating body, growing in magnitude with θ_p_ and *f* (Figure 3G). $\bar{F_{a}}$ is positively related to θ_p_ and *f*, as it is proportional to the acceleration. Incorporating the above information, a larger θ_p_ generally leads to greater *F*_w_, *F*_a_, and $\bar{F_{c}}$, resulting in higher total thrust forces and faster swimming speeds (Figure 3J). The growth rate of the average swimming speed ($\bar{U}$) is notably steeper at larger θ_p_ values. Remarkably, at θ_p_ = 60°, propulsion becomes feasible even at a low *f* of 20 Hz. For diminished θ_p_ values, propulsion emerges at elevated *f* levels.

Focusing on energy, the work done by magnetic torque distributes in interacting with the water and in being stored within the elastic body. The maximum elastic energy of the body (*E*_elastic_, Experimental Section: Estimation of the elastic energy) increases with both θ_p_ and *f*, mirroring the observed trend in body curvature (Figure 3H). Similarly, the work done by magnetic torque in one cycle (*E*_magnetic_, Experimental Section: Estimation of efficiency) follows this trend, with higher energy expenditures leading to faster swimming speeds (Figure 3I). However, despite these differences in energy consumption, the cost of transport (COT) across varied θ_p_ remains close, showing a general increase with *f* but featuring several local minima, resulting in a non‐linear, wavy profile (Figure 3K).

### 2.4 Influence of the Body Stiffness

**Figure 4:**
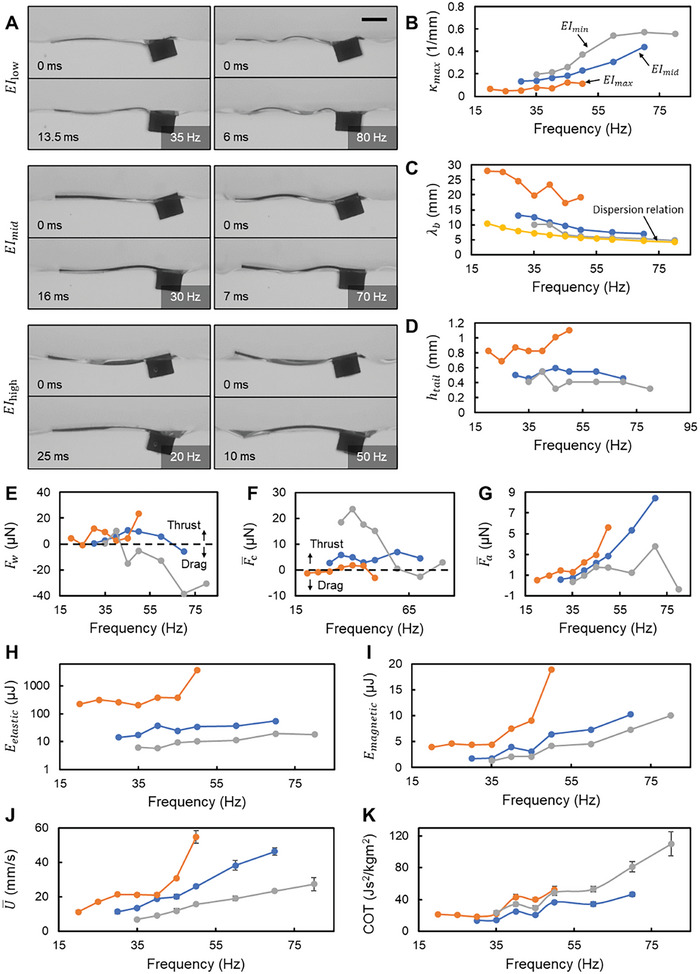
The influence of varying the bending stiffness, *EI*. A) The video snapshots of the swimmers with different *EI* from the side view. The scale bar is 2 mm. B–D) Body waveform parameters, including the maximum curvature at the middle part of the body, κ_max_, the average wavelength of the body waveform, λ_b_, and the peak‐to‐peak amplitude of the tail tip, *h*_tail_. E–G) Forces acting on the swimmer during propulsion, including the wave‐induced force, *F*_w_, the time‐averaged capillary forces along *x* direction, $\bar{F_{c}}$, and the time‐averaged forces due to added mass, $\bar{F_{a}}$. H,I) The maximum elastic energy due to the body's bending, *E*_elastic_, and the work done by magnetic torque in one cycle, *E*_magnetic_. In (H), the vertical axis has a logarithmic scale. J,K) The average swimming speed, $\bar{U}$, and cost of transport, *COT*. In (B–I), the value of each data point is calculated from the body profile extracted from one period. In (J,K), the value of each data point is averaged from five independent trials, and the error bar represents the standard deviation. The error bars on some points are invisible due to the small standard deviation.

As the second parameter, we varied *EI* (*EI*_low_ = 1.26 × 10^−11^ Nm^2^, *EI*_mid_ = 1.83 × 10^−10^ Nm^2^, *EI*_high_ = 8.77 × 10^−9^ Nm^2^) while fixing *L* = 10 mm and θ_p_ = 26°. Sideview video snapshots of the body kinematics at differing *f* and *EI* values are presented in **Figure** **4**A and Movie S3 (Supporting Information). Swimmers with a lower *EI* display a wavier body waveform, which is quantified by the κ_max_ values in Figure 4B. With the exception of the *EI*_low_ swimmer, κ_max_ generally rises with *f*. For the *EI*_low_ swimmer, κ_max_ increases first and then plateaus post 60 Hz. When observing λ_b_, high‐stiffness body swimmers noticeably deviate from λ_d_. Conversely, the *EI*_low_ swimmer's λ_b_ aligns closely with λ_d_, indicating that hydrodynamic forces predominantly shaped its body waveform over its inherent body bending stiffness (Figure 4C). A positive correlation exists between *h*_tail_ and *EI*, signifying that the wave behind the *EI*_high_ swimmer generates the largest thrust (Figure 4D).

The variation of *F*_w_ (Figure 4E), which takes the front wave into consideration, shares a similar trend as *h*_tail_. Although the values of *h*_tail_ differ between *EI*_high_ and *EI*_mid_, the values of *F*_w_ of these two groups are close, which is a result of a considerable drag induced by the front wave of the *EI*_high_ swimmer due to its high speed (Figure 4J). The small *h*_tail_ value of the *EI*_low_ swimmer even turns *F*_w_ to negative. The pronounced curvature of the *EI*_low_ swimmer leads to the maximum $\bar{F_{c}}$, whereas the *EI*_high_ swimmer with small curvature exhibits negligible $\bar{F_{c}}$ (Figure 4F). The thrust caused by the added mass of *EI*_low_ was diminished, particularly at higher actuation frequencies, a stark contrast to its *EI*_mid_ and *EI*_high_ counterparts (Figure 4G). This is because the bending stiffness of the *EI*_low_ swimmer is too low to fight against the hydrodynamic forces. The propulsion speed, $\bar{U}$, evidently depends on bending stiffness: an elevated *EI* corresponds to a higher $\bar{U}$, and its rate of increase with respect to *f* also increases (Figure 4J). It should be noted that the swimmer with a higher *EI* can achieve propulsion at lower *f* levels.

Although the curvature of *EI*_high_ is less than that of *EI*_mid_ and *EI*_low_, its *E*_elastic_ exceeds the others by 1–2 orders of magnitude (Figure 4H). This results in a heightened *E*_magnetic_ (Figure 4I) and COT (Figure 4K), indicating reduced efficiency. While the *EI*_low_ swimmer achieves the least *E*_elastic_ and *E*_magnetic_, its COT surpasses that of the *EI*_mid_ due to a reduced $\bar{U}$. This emphasizes that extreme bending stiffness, whether excessively high or low, can compromise propulsion efficiency.

### 2.5 Influence of the Body Length

**Figure 5:**
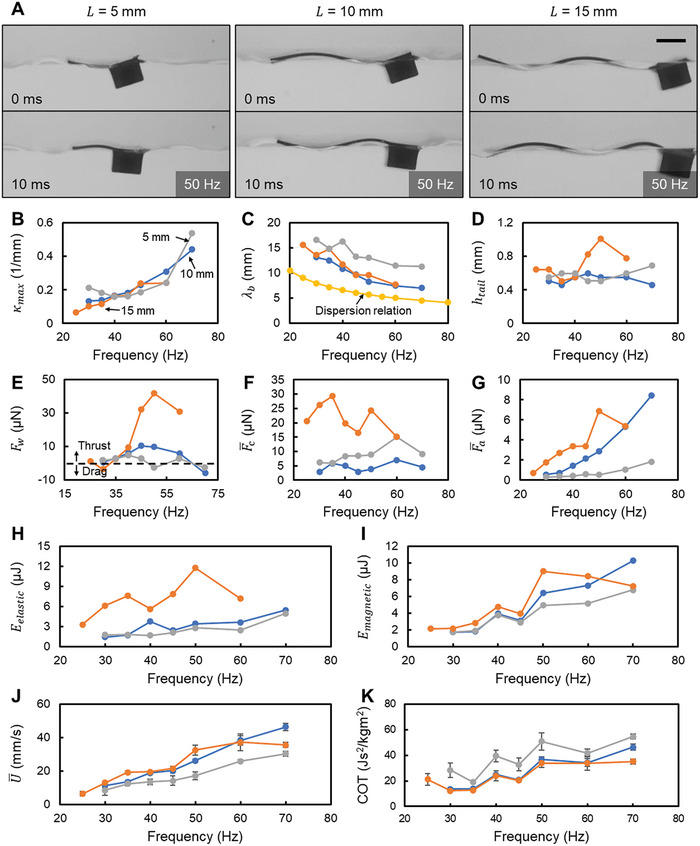
The influence of varying the body length, *L*. A) The video snapshots of the simmers with different *L* from the side view. Scale bar is 2 mm. B–D) Body waveform parameters include the maximum curvature at the middle part of the body, κ_max_, the average wavelength of the body waveform, λ_b_, and the peak‐to‐peak amplitude of the tail tip, *h*_tail_. E–G) Forces acting on the swimmer during propulsion include the wave‐induced force, *F*_w_, the time‐averaged capillary forces along *x* direction, $\bar{F_{c}}$, and the time‐averaged forces due to added mass, $\bar{F_{a}}$. H,I) The maximum elastic energy due to the body's bending, *E*_elastic_, and the work done by magnetic torque in one cycle, *E*_magnetic_. J,K) The average swimming speed, $\bar{U}$, and cost of transport, *COT*. In (B–I), the value of each data point is calculated from the body profile extracted from one period. In (J,K), the value of each data point is averaged from five independent trials, and the error bar represents the standard deviation. The error bars on some points are invisible due to the small standard deviation.

As the third parameter, we fixed θ_p_ = 26 and *EI* = 1.83 × 10^−10^ Nm^2^, while varying *L* (5, 10, and 15 mm). **Figure** **5**a and Movie S4 (Supporting Information) showcase the swimming kinematics across the three conditions. Notably, the variation in *L* exerted a minimal impact on κ_max_ (Figure 5B). Predominantly, the body waveforms hinge on the body's bending stiffness rather than hydrodynamic forces, evidenced by the marked difference between λ_b_ and λ_d_ (Figure 5C). The implications of adjusting *L* on both λ_b_ and *h*_tail_ appear multifaceted. The λ_b_ values for *L* = 10 and 15 mm closely align, whereas the λ_b_ for *L* = 5 mm is discernibly higher across all frequency ranges. *h*_tail_ remains consistent for *L* = 5 mm and 10 mm across the frequency spectrum, yet for *L* = 15 mm, while being on par with the other lengths below 40 Hz, it surpasses them at elevated frequencies (Figure 5D).

As *F*_w_ and $\bar{F_{a}}$ scale with *L* ^3^, and $\bar{F_{c}}$ scales with *L* ^2^, increasing the swimmer's length can effectively enhance the thrust. The data presented in Figure 5E indicate that *F*_w_ correlates with the trend of *h*_tail_, with the *L* = 15 mm swimmer achieving the highest thrust from the wave at frequencies above 40 Hz, attributed to its relatively high *h*_tail_. Furthermore, this *L* = 15 mm swimmer generates the greatest capillary force, benefiting from an enlarged contact surface, as shown in Figure 5F. Figure 5G reveals a length‐dependent trend for $\bar{F_{a}}$: swimmers with elongated bodies register higher $\bar{F_{a}}$ values.

Figure 5J displays average speeds for all conditions, with *L* = 10 mm and *L* = 15 mm swimmers recording very close $\bar{U}$ values. This observation appears to contradict the fact that the *L* = 15 mm swimmer generates the greatest thrust. Given that the swimmers were tested in a narrow channel (Experimental Section: Body profile extraction and waveform parameter calculation), it is plausible that the increased body length contributes to greater friction when the swimmer comes into contact with the channel walls. On the other hand, the shortest‐length swimmer lags in speed across all frequencies. All swimmers exhibit comparable $\bar{U}$ growth rates concerning *f*. Analyzing energy consumption, the *L* = 15 mm swimmer expends more energy in elastic body bending across frequencies up to 50 Hz (Figure 5H), but its *E*_magnetic_ decreases post 50 Hz, even lowering than *L* = 10 mm after 60 Hz (Figure 5I). The COT values for *L* = 10 mm and *L* = 15 mm hover closely, but *L* = 5 mm emerges as the least efficient (Figure 5K).

### 2.6 Particle Image Velocimetry

While the preceding analysis was conducted within a 2D framework, it is important to note that the flow field surrounding the swimmer is inherently 3D. To examine the flow field around the swimmer during propulsion, we employed particle image velocimetry (PIV) to visualize flow on different planes (Experimental Section: Particle image velocimetry).

**Figure 6:**
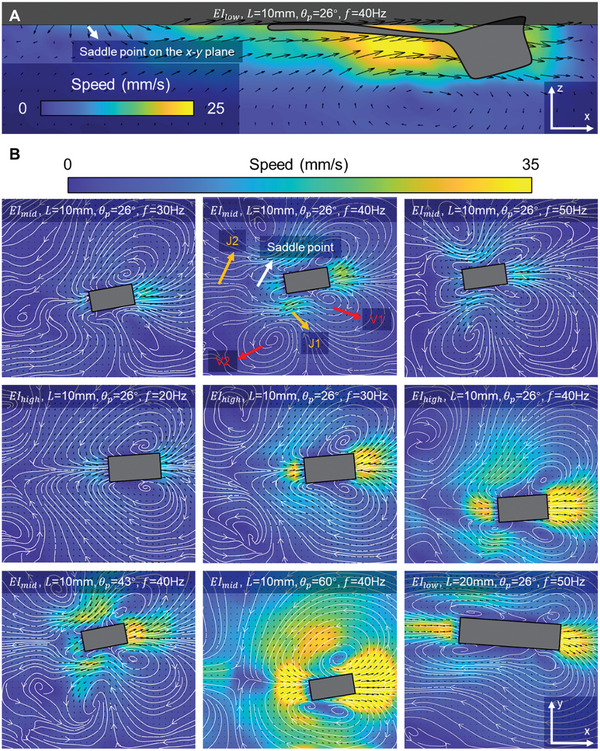
Investigation of the flow fields around the swimmer using the particle image velocimetry. A) 2D flow field observed from the side view. B) 2D flow fields on the water surface observed from the top view. The flow fields are calculated by averaging instantaneous flow fields in one cycle.

On the *x*‐*z* plane, we notice that the fluid disturbances were less in the deeper regions compared to fluid regions closer to the surface. This is illustrated by the time‐averaged velocity field in **Figure** **6**A. Notably, fluid ≈1.5 mm deep from the water surface experienced pronounced acceleration, aligning with the size of the swimmer's head. This phenomenon is attributed to the swimmer's head's forward movement, which creates a low‐pressure region and causes adjacent fluids to move in sync. This movement is not a direct result of the undulatory motion of the soft body, prompting us to further investigate the flow information on the water surface.

The flow fields on the water surface were reconstructed by observing the movement of microparticles on the interface (Movie S1, Supporting Information). For the swimmer with *EI*_mid_, *L* = 10 mm, and θ_p_ = 26°, the time‐averaged flow fields presented in the first row of Figure 6B display a “butterfly” structure. Here, two leading‐edge vortices (V1) attach to the swimmer's forefront, with another vortex pair (V2) forming in its wake. A portion of the fluid is pushed forward by the swimmer's head, while behind the body, a portion of fluid chases the swimmer. The V1‐generated flows create side‐backward jets (J1), while the V2‐surrounding flows result in a rearward jet (J2) of lesser magnitude. This J2 jet, combined with the flow chasing the swimmer, forms a distinguishable saddle point, which is also observable in the flow field on the *x*‐*z* plane. At a frequency of 30 Hz, this butterfly‐like structure is not fully developed, with only the V1 vortices prominently visible. But as the frequency rises to 40 and 50 Hz, the V2 vortices emerge, accompanied by increased jet flow velocity.

Moving on to the swimmer with *EI*_high_, *L* = 10 mm, and θ_p_ = 26°, depicted in Figure 6B's second row, V1 and V2 vortices are more separated, and the V1 vortex zone expands, causing J1 flows to converge inward. Notably, even at lower frequencies, the presence of both V1 and V2 vortices is clearly distinguishable, and the flow velocity magnitude is stronger, which contrasts to *EI*_mid_ case.

Adjusting θ_p_ also elicited significant flow changes (the first two panels in the last row of Figure 6B). A modest rise in θ_p_ to 43° primarily tweaked jet magnitudes without altering the flow structure. Conversely, elevating θ_p_ to 60° initiated a notable structural change, characterized by expanded area and backward shift of V1 vortices and inward J1 — a transformation reminiscent of the shifts observed with higher *EI*.

With *L* expanded to 20 mm (shown in the final panel of Figure 6B), the V2 vortices clung onto the swimmer's body, merging with V1 vortices. This configuration steered J1 to flow more in parallel with the swimmer's body. The streamlines also suggest that a portion of fluids escape from the body's midsection sideward.

## 3 Discussion

### 3.1 Undulatory Locomotion on Water Surface

A notable observation in diverse biological systems is the ubiquitous undulatory locomotion, which spans environments from aquatic to terrestrial and length scales from micrometers to meters.^[^ ^15^ ^]^ This locomotion can be actively driven by coordinated muscle contractions, passively induced by external forces,^[^ ^16^ ^]^ or arise from a combination of both mechanisms.^[^ ^17^ ^]^ The prevalence of the undulatory motion prompts the hypothesis that undulatory movements may offer inherent mechanical and fluid dynamic advantages that make them a favored mode of locomotion. In low Reynolds number (Re) conditions, where viscous forces dominate, undulatory movements circumvent the constraints of the Scallop theorem^[^ ^18^ ^]^ by breaking spatially symmetric reciprocal motion to achieve net propulsion. In high Re conditions, where inertial forces are dominant, undulatory movements efficiently transmit momentum backward, as described by elongated‐body theory,^[^ ^19^ ^]^ thereby generating forward thrust through vortex formation and jet streams. On terrestrial terrains, animals like snakes leverage the undulatory sidewinding motion to enable directed crawling on sand surfaces that are yielding and slippery.^[^ ^15b^ ^]^

Our study underscores the viability of undulatory motion for achieving effective propulsion on the water surface. Such a movement is initiated by introducing a pitch motion to the leading edge of a flexible sheet. The emergent waveform is a product of both hydrodynamic forces and the intrinsic bending stiffness of the sheet. Previous research exploring the thrust of a vibrating hydrofoil on the water surface emphasized the momentum transferred to the fluid via capillary waves.^[^ ^10^, ^11^ ^]^ Our research revealed that solely characterizing the wave's radiation stress is insufficient, as wave‐induced forces varied among swimmers with similar swimming speeds. Therefore, it's essential to consider the hydrodynamic forces stemming from the direct interaction between the swimmer and the surrounding fluids. We addressed this by calculating the force due to added mass along the body utilizing the information from the body kinematics. We specifically incorporate the added mass in our analysis as it offers a reasonable estimate of the hydrodynamic force acting on an object rapidly lifted from the water surface, especially during the initial stages.^[^ ^13^ ^]^ Additionally, the undulatory movement deforms the water surface. This deformation is significant for inducing capillary force‐induced thrust or drag, which is comparable in magnitude to the thrust generated from both the wave and added mass.

Theoretically, this propulsion mechanism is adaptable to interfaces beyond the air‐water boundary. Employing different fluids to form the interface alters the fluid properties and interfacial tension, consequently modifying the swimmer's waveform, interfacial wave propagation,^[^ ^20^ ^]^ and the resultant hydrodynamic forces. The dynamic interaction between the flexible swimmer and the dual liquid phases constitutes a complex system. A detailed quantitative analysis is requisite for a comprehensive understanding of how each parameter influences the overall propulsion strategy.

### 3.2 Implications to Designing Miniature Swimming Robots on Water Surface

Actuating a flexible hydrofoil via vibration at the leading edge is a promising approach for developing miniature robots designed to navigate on water surfaces. Optimizing control parameters, such as head excitation amplitude and frequency, combined with the careful design of flapping fin/tail properties like bending stiffness and length, can facilitate both rapid and energy‐efficient propulsion. Increasing the values of parameters such as frequency (*f*), pitch angle amplitude (θ_p_), and bending stiffness (*EI*) can enhance swimming speeds through augmentation of wave‐induced force (*F*_w_), added mass force ($\bar{F_{a}}$) or capillary force ($\bar{F_{c}}$). Although all these strategies result in higher energy consumption, an increase in θ_p_ does not significantly hamper the swimming efficiency measured in COT, which contrasts with elevating *f* and *EI*. A short *L* negatively impacts both $\bar{U}$ and COT, as *F*_w_, $\bar{F_{c}}$, and $\bar{F_{a}}$ are all hampered. However, extending *L* excessively does not offer commensurate benefits in terms of COT and $\bar{U}$, despite the observed increases in *F*_w_, $\bar{F_{c}}$, and $\bar{F_{a}}$. This phenomenon is likely due to the elevated friction encountered when the swimmer contact with the channel walls, which counteracts the benefits of increased thrust forces. In addition, increasing θ_p_, *EI*, and *L* can lower the frequency threshold of propulsion, which is important for actuators that cannot reach high vibration frequencies.

It is worth noting that undulatory motion is not a prerequisite for surface propulsion. As Figure S1 (Supporting Information) illustrates, a relatively rigid body can still exploit the asymmetry in radiation pressure to achieve thrust. In the absence of an undulatory waveform, $\bar{F_{a}}$ and $\bar{F_{c}}$ become negligible in comparison to *F*_w_, which is orders of magnitude greater (Figure S1C, Supporting Information). This observation corroborates the thrust estimation method for a surface swimmer with a stiff body discussed in the previous study.^[^ ^11c^ ^]^ The primary advantage of undulatory motion lies in reducing the actuation energy by decreasing the energy needed to bend the body and the volume of fluid accelerated during swimming. A judicious design choice of *EI* can strike a balance between energy consumption and swimming speed, resulting in optimal propulsion efficiency. This consideration is especially crucial for miniature swimmers, which are limited in terms of power output and onboard energy reserves.

While undulatory motion offers this advantage in water surface propulsion, it may also introduce a downside: increased drag due to water surface deformation. This aspect is notably different from undulatory movements inside water. Although our observations suggest that capillary forces sometimes hinder propulsion, they also present an opportunity to improve the load‐bearing capacity on the water surface,^[^ ^21^ ^]^ which is particularly relevant for boosting the cargo‐carrying capabilities of miniature swimming robots, whose small size limits their ability to generate larger buoyant forces.

### 3.3 Influence of Scaling

When all dimensions of the swimmer, including its length, width, and thickness, are reduced, its bending rigidity decreases following an L^4^ scale, whereas capillary forces and added mass forces scale with L^2^ and L^3^, respectively. This differential scaling implies that as the swimmer's size diminishes, its flexible body increasingly struggles to counteract hydrodynamic forces. Consequently, the relative influence of the swimmer's bending moment wanes in comparison to hydrodynamic forces, and its body waveform may align with that of capillary waves on a free water surface. Under these conditions, time‐averaged forces nullify due to the symmetric oscillation amplitudes of the capillary waves.^[^ ^22^ ^]^ Moreover, in the realm of low Reynolds numbers, viscous forces predominate over inertial forces to such an extent that energy dissipation prevents wave propagation and, thus, the generation of wave‐induced forces. Therefore, the propulsion becomes progressively challenging as the swimmer decreases in size.

In such cases, enhancing the actuation frequency proves beneficial, enabling the robot to achieve greater thrust. A higher actuation frequency leads to increased body curvatures, accelerated fluid movements, and larger water wave numbers, collectively amplifying thrust through augmenting *F*_c_, *F*_a_, and *F*_w_. This adaptation, leveraging higher actuation frequencies for propulsion at low Reynolds numbers, is a strategy observed in numerous small‐scale aquatic organisms. For instance, adult zebrafish typically swim within a frequency range of 1–21 Hz.^[^ ^23^ ^]^ In contrast, larval zebrafish exhibit beating frequencies as high as 100 Hz,^[^ ^24^ ^]^ illustrating the biological precedent for this mechanical principle.

As the swimmer increases in size, buoyancy becomes a critical limiting factor. The floating force provided by the water surface is derived from buoyancy, as per Archimedes' principle, and the normal component of the surface tension force. Alternatively viewed, the buoyant force equates to the weight of the displaced water by the object's submerged volume and the meniscus at the contact line.^[^ ^25^ ^]^ A simple calculation indicates that the swimmer could be scaled up to ≈2.3 times its current dimensions, equivalent to 257 mg, without risking submersion in a static state (Text S1, Supporting Information). However, the assumption of a static state does not accurately reflect the dynamic conditions experienced during propulsion. Excessive pitch angle amplitudes and actuation frequencies may cause the swimmer, even at its current size, to sink due to surface disruption. The exact maximum scaling factor under dynamic conditions is a complex function of both the actuation signal and the dimensional design, warranting further detailed investigation. Nonetheless, the preliminary analysis provides a valuable upper limit for estimating the swimmer's maximum dimension.

### 3.4 Limitations of This Work

Our assessments of *F*_w_, $\bar{F_{a}}$, and $\bar{F_{c}}$ were grounded in a 2D framework, which might not accurately represent the inherent complexities of the 3D world. Notably, capillary waves originating from the swimmer disperse omnidirectionally (Figure 2A), implying that wave‐induced forces are not only oriented in the propulsion direction. Additionally, under certain conditions, water might escape laterally from the swimmer's long edge (Figure 6B), potentially contributing additional thrust. Furthermore, the force calculations hinge on discretized body profiles extracted from one period. Factors like the accuracy of the body profile extraction and the choice of the time instance can affect the accuracy of these calculations. While this study touched upon the fluid structure around the swimmer, a deeper exploration is required. The intricate interplay between flow structure and swimming performance presents a rich area for detailed future research.

## 4 Experimental Section

### Design and Fabrication of Milliswimmers

Figure S2 (Supporting Information) illustrates the milliswimmer's design, which was fabricated based on the method outlined in our previous study.^[^ ^26^ ^]^ The head of the swimmer is composed of polydimethylsiloxane (PDMS, Sylgard 184, Dow Corning GmbH), with a 10:1 monomer to crosslinking agent ratio. A cubic magnet with a 1 mm side length (W‐01‐N Supermagnete, Webcraft GmbH) was embedded in the square cavity of the swimmer's head and sealed with Ecoflex 0010. The flexible sheet was made of either PDMS or Ecoflex 0010 (Smooth‐On, Inc). The bodies of *EI*_mid_, *EI*_low_ swimmers were made of Ecoflex 0010 with thicknesses of 200 and 82 µm, respectively. The body of *EI*_high_ swimmer was made of PDMS with a thickness of 200 µm. Other swimmers were all made of Ecoflex 0010 with a thickness of 200 µm.

### Body Profile Extraction and Waveform Parameter Calculation

To extract the body profiles, the swimmers were confined in an 8 mm × 100 mm channel. Their body movements were recorded using a high‐speed camera with an acquisition rate of 2000 fps from the side view. To extract the body profile at a certain frame, first digitized marker points were placed along the soft body in the image, and then cubic Hermite interpolating polynomial was used piecewise to get a fitted curve from these points. The fitted curve was resampled to get 200 uniformly spaced points. For each trial, *N*_f_ = 10–12 frames with uniform time intervals were extracted from one period.

The curvatures at the middle locations (indicated by the red dashed line in Figure 3A) of the profiles are calculated, and the maximum curvature obtained from the *N*_f_ profiles was reported as κ_max_. For a discretized profile, the curvature κ_mid_ at the middle point (*x*_m_, *y*_m_) can be calculated as:

(1)

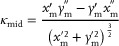

where the prime and the double prime represent the first and the second derivatives with respect to the curvilinear coordinate of the profile. To calculate λ_b_, we use the formula with the following form to fit profiles:

(2) $$y\;=\left(p_{1}+p_{2}x\right)\;\sin\left(2\pi p_{3}x+p_{4}\right)+p_{5}$$

where *p*_i_ is the fitting parameters, and the wavelength of the profile being fitted is 1/*p*_3_. The average of 1/*p*_3_ of all *N*_f_ profiles are reported as λ_b_. The value of *h*_tail_ was obtained by measuring the peak‐to‐peak amplitude of the swimmer's tail tip from the videos.

### Surface Wave Reconstruction

The waves on the water surface were reconstructed following the Schlieren technique detailed in ref. [27] The checkerboard pattern with a pixel size of 0.5 mm was printed using an inkjet printer (CX924 dxe, Lexmark) on white paper. The pattern was put under a glass tank with a wall thickness of 3 mm. It was assumed that there was no gap between the checkerboard pattern and the tank. Under the pattern was a white LED panel for illumination. A high‐speed camera was arranged above the tank to capture the swimmer's propulsion from the top view. The Schlieren imaging requires the camera to focus on the checkerboard pattern at the tank bottom. To ensure the boundaries of the swimmer can be clearly distinguished from the videos, the water depth was set to 5 mm. The videos were analyzed through an open‐source code.^[^ ^27a^ ^]^

### Estimation of the Wave‐Induced Force

The wave‐indued force was calculated using the amplitude and wavelength of the wave in front and behind the swimmer along the body's midline. In deep water (*kH* ≫ 1, where *k*  = 1/λ  is the wavenumber and *H* is the water depth), the radiation stress due to the capillary‐gravity wave can be calculated by^[^ ^14^ ^]^.

(3) $$S\;=\frac{3}{4}\;\gamma h^{2}k^{2}$$

where γ is the surface tension of water, and *h* is the amplitude of the wave. With the 2D assumption, the thrust force results from the radiation stress of the water wave is:

(4) $$F_{w}=\left(S_{b}-S_{f}\right)\;w$$

where *S*_r_ and *S*_f_ are the radiation stresses behind and in front of the swimmer, and *w* is the swimmer's width. The *F*_w_ data provided in Figure 2D were calculated using the wave parameters obtained through Schlieren imaging, while the *F*_w_ data in Figures 3, 4, 5 were derived from amplitudes and wavelengths directly measured from the side‐view high‐speed camera images.

### Estimation of the Force Due to Added Mass

The force due to added mass was calculated from the body profiles with a 2D assumption. According to a linearized model of water exit,^[^ ^13^ ^]^ the hydrodynamic force can be modeled as the added mass times the acceleration. The force due to added mass acting on the segment between adjacent digitized points was calculated by

(5) $$F_{a}\;\left(s\right)=\frac{\rho \pi w{\left(ds\right)}^{2}}{8}\;a\left(s\right)$$

where *a*(*s*) is the acceleration of the body segment at the coordinate *s*, and ρ is the water density at room temperature. ref. [28] was referred to get the added mass coefficient. The total force due to the added mass along the propulsion direction in the frame *i* is calculated by summing up the *x*‐component of *F*_a_(*s*) along the body. The time‐averaged force due to added mass is calculated by

(6) $$\bar{F_{a}}=\frac{\sum_{i=2}^{N}\left(F_{a}\left(i-1\right)+F_{a}\left(i\right)\right)}{2\left(N_{f}-1\right)}\;$$

### Estimation of the Capillary Force

The capillary force was calculated from the body profiles with a 2D assumption. The curvature κ(*s*) along the soft body was calculated using the body profiles extracted from the videos. Here, *s* is the curvilinear coordinate. The pressure difference across the interface at the point of interest was calculated through

(7) $$\Delta P\left(s\right)\;=\gamma_{\mathrm{sl}}\;\kappa \left(s\right)$$

where γ_sl_ is the interfacial tension between the soft body and water. Δ*P* is always positive as the pressure of the water phase is always larger than the air phase. The pressure difference across the interface induces a capillary force normal to the body profile:

(8) $$F_{c}\;\left(s\right)=\;w\Delta P\left(s\right)ds$$

where *ds* is the distance between adjacent points on the body profile, and *w*  =  5 *mm* is the body width. The total capillary force along the propulsion direction in the frame *i* is calculated by summing up the *x*‐component of *F*_c_(*s*) along the body. The time‐averaged capillary force is calculated by

(9) $$\bar{F_{c}}=\frac{\sum_{i=2}^{N}\left(F_{c}\left(i-1\right)+F_{c}\left(i\right)\right)}{2\left(N_{f}-1\right)}\;\;$$

### Measuring the Interfacial Tension Between Water and Soft Materials

The interfacial tension between water and soft materials was calculated by fitting the Owens, Wendt, Rabel, and Kaelble (OWRK) model:

(10) $$\frac{\gamma_{l}\left(1+cos\Theta \right)}{2\sqrt{\gamma_{l}^{d}}}=\sqrt{\gamma_{s}^{p}}\;\cdot \sqrt{\frac{\gamma_{l}^{p}}{\gamma_{l}^{d}}}+\sqrt{\gamma_{s}^{d}}$$

where the subscripts *l* and *s* represent, respectively, the liquid and solid phases, and the superscripts *p* and *d* represent respectively the dispersed and polar components of the liquid being tested. The contact angles, Θ, of the liquids with known surface tension, γ_*l*_, and disperse and polar components of the surface tension were measured using the sessile drop technique. The data are shown in Figure S3 (Supporting Information), where, the *x*‐axis is $\sqrt{\gamma_{l}^{p}}$, and the *y* axis is $\frac{\gamma_{l}\left(1+cos\Theta \right)}{2\sqrt{\gamma_{l}^{d}}}$. A line was fitted using the least square regression. $\gamma_{s}^{p}$ and $\gamma_{s}^{d}$ can then be calculated from the slope and intercept of the fitted line. The interfacial tension between the solid and liquid phases can then be calculated by:

(11) $$\gamma_{sl}=\gamma_{s}^{p}\;+\gamma_{s}^{d}+\gamma_{l}-2\sqrt{\gamma_{s}^{d}\gamma_{l}^{d}}-2\sqrt{\gamma_{s}^{p}\gamma_{l}^{p}\;}$$

During the experiments, three liquids were tested with known surface tension values on substrates made of Ecoflex 0010 and PDMS.

### Estimation of the Elastic Energy

The elastic energy of the soft body was calculated from the body profiles through

(12) $$E_{\mathrm{elastic}}=\frac{1}{2}\;\int_{0}^{L}EI\kappa {\left(s\right)}^{2}ds$$

where *L* is the length of the soft body, and *EI* is the bending stiffness. The maximum value of *E*_elastic_ is reported in the main text.

### Estimation of Efficiency

The magnetic energy consumed in one beating cycle was calculated through

(13) $$E_{\mathrm{magnetic}}=\int_{0}^{T}MB\left(t\right)cos\theta \left(t\right)dt\;$$

where *M* is the magnetization magnitude of the permanent magnet, *B*(*t*) is the magnetic field at time *t*, θ(*t*) is the angle between the magnetic field and the magnetization, and *T* is the time duration of one cycle. The magnetization of the permanent magnet was determined using a vibrating sample magnetometer (EZ7, Microsense). The cost of transport was calculated through

(14) $$COT\;=\frac{E_{\mathrm{magnetic}}}{mgd}\;$$

where *m* is the weight of the swimmer, *g* is the standard acceleration of gravity, and *d* is the travel distance in one cycle. A high *COT* represents a low energy efficiency, while a low *COT* implies a high energy efficiency, and vice versa.

### Particle Image Velocimetry

Micro glass particles with an average diameter between 45 and 53 µm (Cospheric LLC) were treated with a plasma cleaner (Tergeo, PIE Scientific LLC) and then evenly seeded on the water surface. A white LED panel was placed under the tank for illumination. A high‐speed camera was placed above the tank to capture the particle movement from the top view with an acquisition frequency of 2000 Hz. The particle images were finally analyzed with OpenPIV.^[^ ^29^ ^]^ The flow fields in Figure 6 were obtained by averaging the velocity fields of all frames within one cycle.

### Statistical Analysis

For each parameter combination, the experiment was conducted five times, and only the steady swimming periods were used for data analysis. In the main text, the average values of *U* and *COT*, along with the standard errors are reported in figures. The trial that yielded a propulsion speed close to the average value was selected and the body's undulatory waveform from a complete cycle was extracted. The obtained body profile was then utilized to analyze the waveform properties (κ_max_, λ_b_, and *h*_tail_), different forces ($\bar{F_{c}}$, $\bar{F_{a}}$), and energy consumption (*E*_elastic_, *E*_magnetic_). To calculate *F*_w_, one more experiment was conducted to reconstruct the water surface wave profile using the Schlieren imaging technique.

## Conflict of Interest

The authors declare no conflict of interest.

## Supporting information

Supporting Information

Supplemental Movie 1

Supplemental Movie 2

Supplemental Movie 3

Supplemental Movie 4

## Data Availability

The data that support the findings of this study are available from the corresponding author upon reasonable request.
