# Supplementary material for: Undulatory Propulsion at Milliscale on Water Surface
Source: Adv Sci (Weinh). 2024 Mar 14;11(19):2309807. doi: 10.1002/advs.202309807 (PMC11109636; doi:10.1002/advs.202309807)
Supplement: Supplementary file 1 — Supporting Information [file ADVS-11-2309807-s003.pdf]

ADVANCED SCIENCE
Open Access

Supporting Information

for *Adv. Sci.*, DOI 10.1002/advs.202309807

Undulatory Propulsion at Milliscale on Water Surface

*Ziyu Ren, Kagan Ucak, Yingbo Yan and Metin Sitti**

Supporting Information for

Undulatory Propulsion at Milliscale on Water Surface

Ziyu Ren[1,2], Kagan Ucak[2], Yingbo Yan[2,3], Metin Sitti[2,4,5*]

[1] School of Mechanical Engineering and Automation, Beihang University, Beijing 100191, China

[2] Physical Intelligence Department, Max Planck Institute for Intelligent Systems, 70569 Stuttgart, Germany

[3] Laboratory for Multiscale Mechanics and Medical Science, SV LAB, School of Aerospace, Xi'an Jiaotong University, Xi'an 710049, China

[4] Institute for Biomedical Engineering, ETH Zurich, 8092 Zurich, Switzerland

[5] School of Medicine and College of Engineering, Koç University, 34450 Istanbul, Turkey

[*] Correspondence to: sitti@is.mpg.de

This PDF file includes:

Text S1

Figures S1 to S4

Legends for Movies S1 to S4

Other supporting materials for this manuscript include the following:

Movies S1 to S4

Text S1: Calculation of the buoyant force

The heaviest part of the swimmer is the magnetic head, characterized by the volume of $l \times w \times h = 5 \times 2 \times 1.5mm$. It has been observed that the failure of the floating typically results from the sinking of the head. So we will primarily focus on analyzing the floating/sinking state of it. Let's assume the head totally submerges under the water surface with the meniscus pinned at four edges (Fig. S4a). In the static state, the total floating force can be estimated as:

$$F = \rho_{water} glwh + 2\gamma l \cos(\Theta) + 2\gamma w \cos(\Theta),$$

where ρ_{water} is the water density, g is the gravitational constant, γ is the surface tension coefficient, and $\Theta = 109.4°$ is the contact angle (Fig. S3). The head is composed of a small cubic magnet with a density of around 7.6 mg/mm^3 and side length of 1 mm, and a polymer shell with a density of around 0.97mg/mm^3. The floating force and the weight of the head in relation to the scaling factor is depicted in Fig. S4b. It shows that the head can scale up to around 2.3 times the current dimension, which corresponds to 257mg, without sinking at the static state.

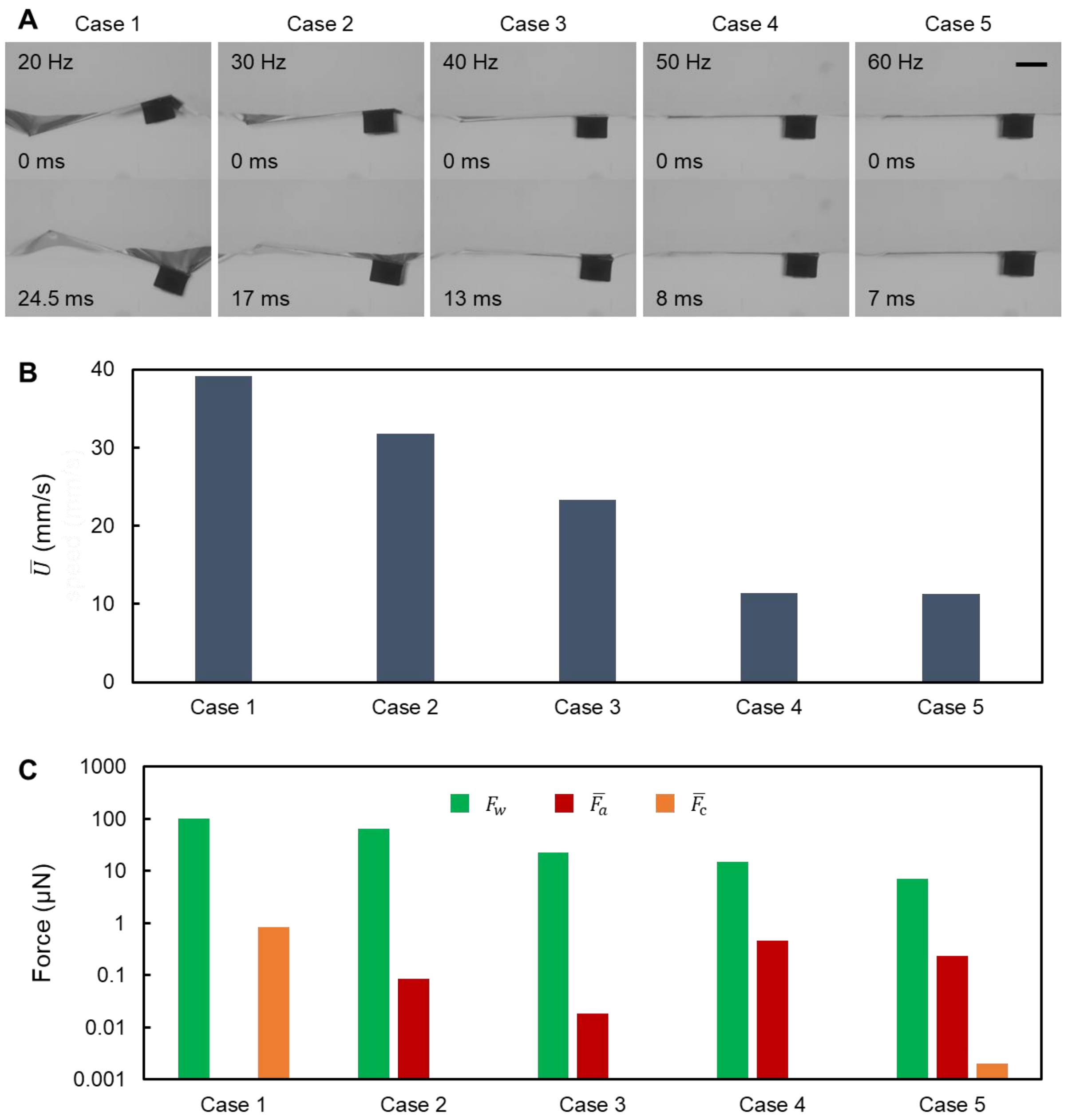

Fig. S1. Characterization of the swimmer with stiff body. (A) The body of the swimmer was made of Mylar film with a thickness of 0.05 mm, resulting in a bending stiffness of around 1.56×10^{-7} Nm^2, which is orders of magnitude larger than the swimmers reported in the main text. We didn't observe a wavy shape even at a very large pitch angle amplitude. Scale bar: 2 mm. (B) The average propulsion speed of the swimmer. (C) The forces arising from the swimming. Note the vertical axis has a logarithmic scale. The force induced by wave is orders of magnitudes larger than the force due to added mass and the capillary force.

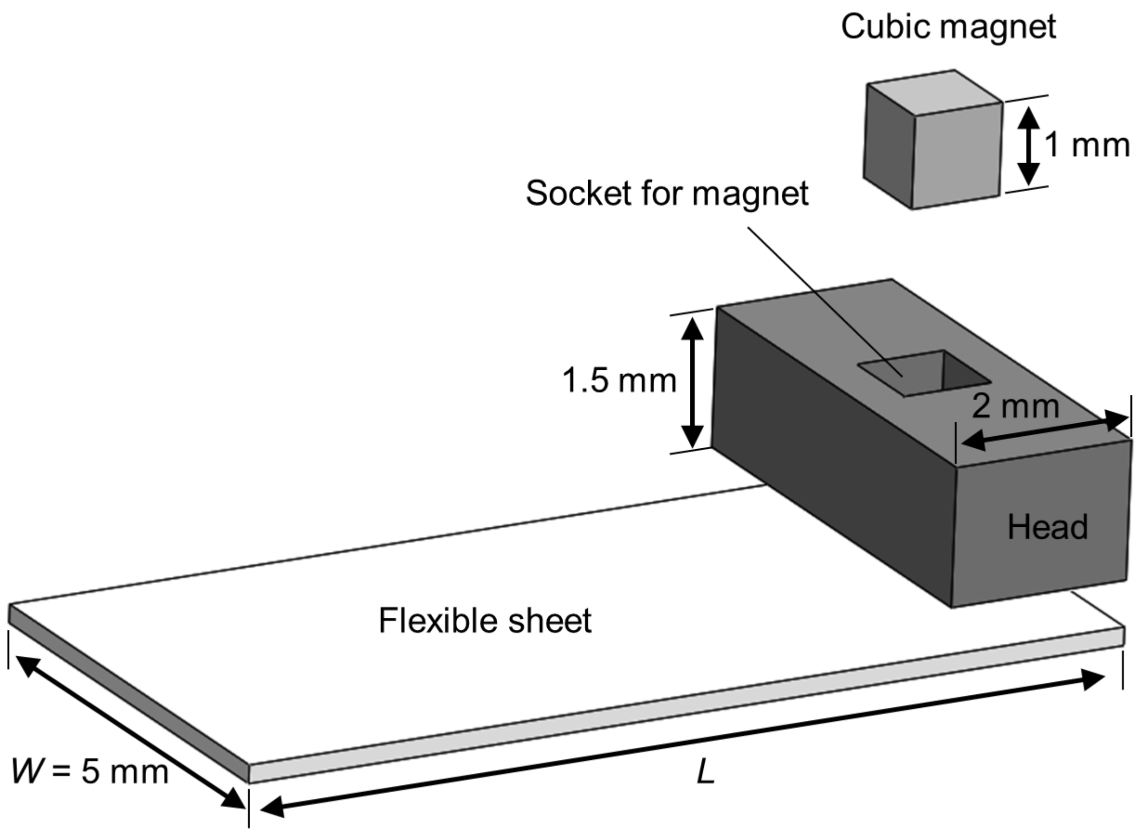

Fig. S2. Design of the milliswimmer.

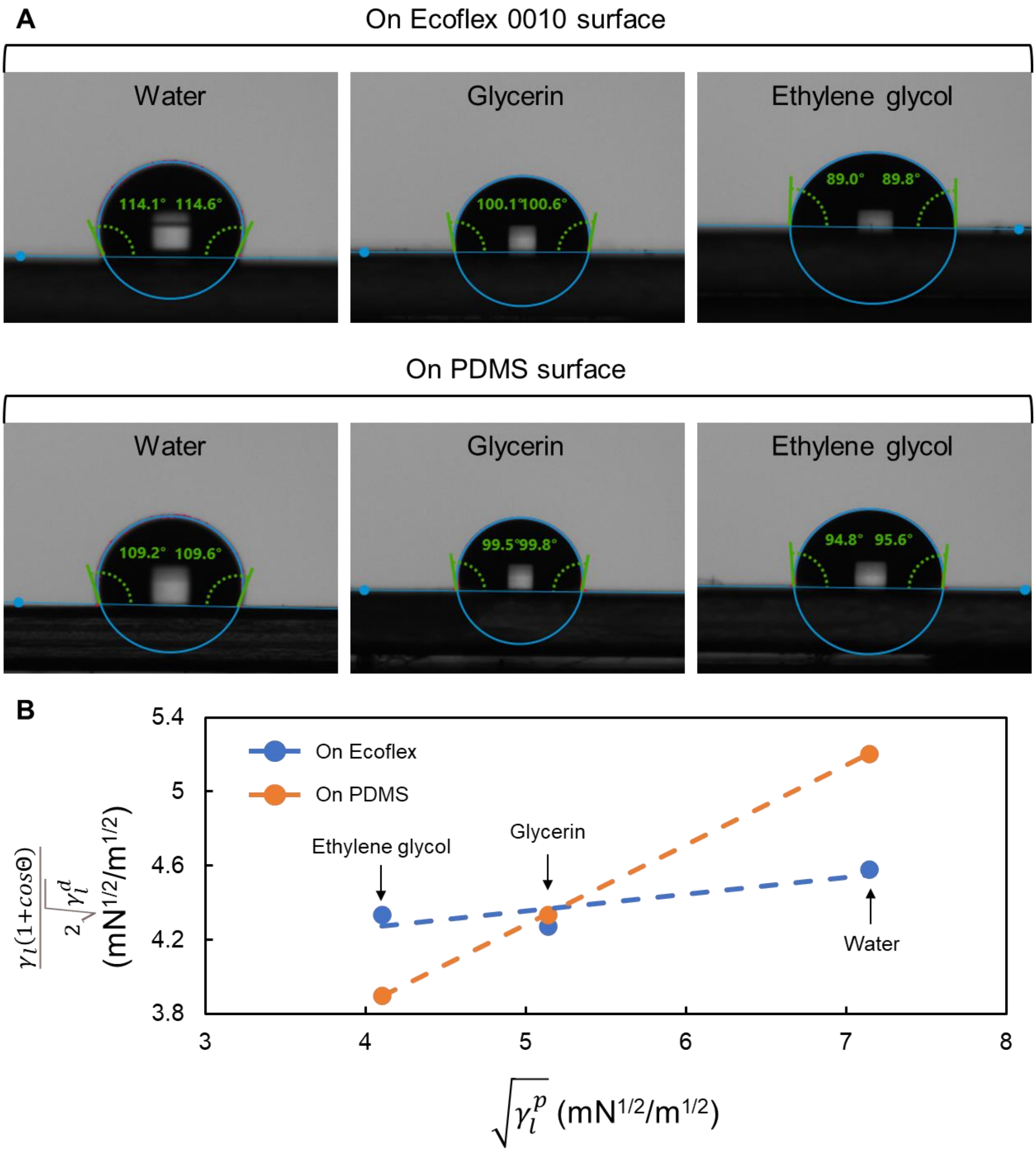

Fig. S3 Contact angle measurement. (A) Sessile drop experiments were conducted on substrates made of Ecoflex 0010 and PDMS. Three liquids with known disperse and polar components of the surface tension were used. (B) The measurement results were linearly fitted.

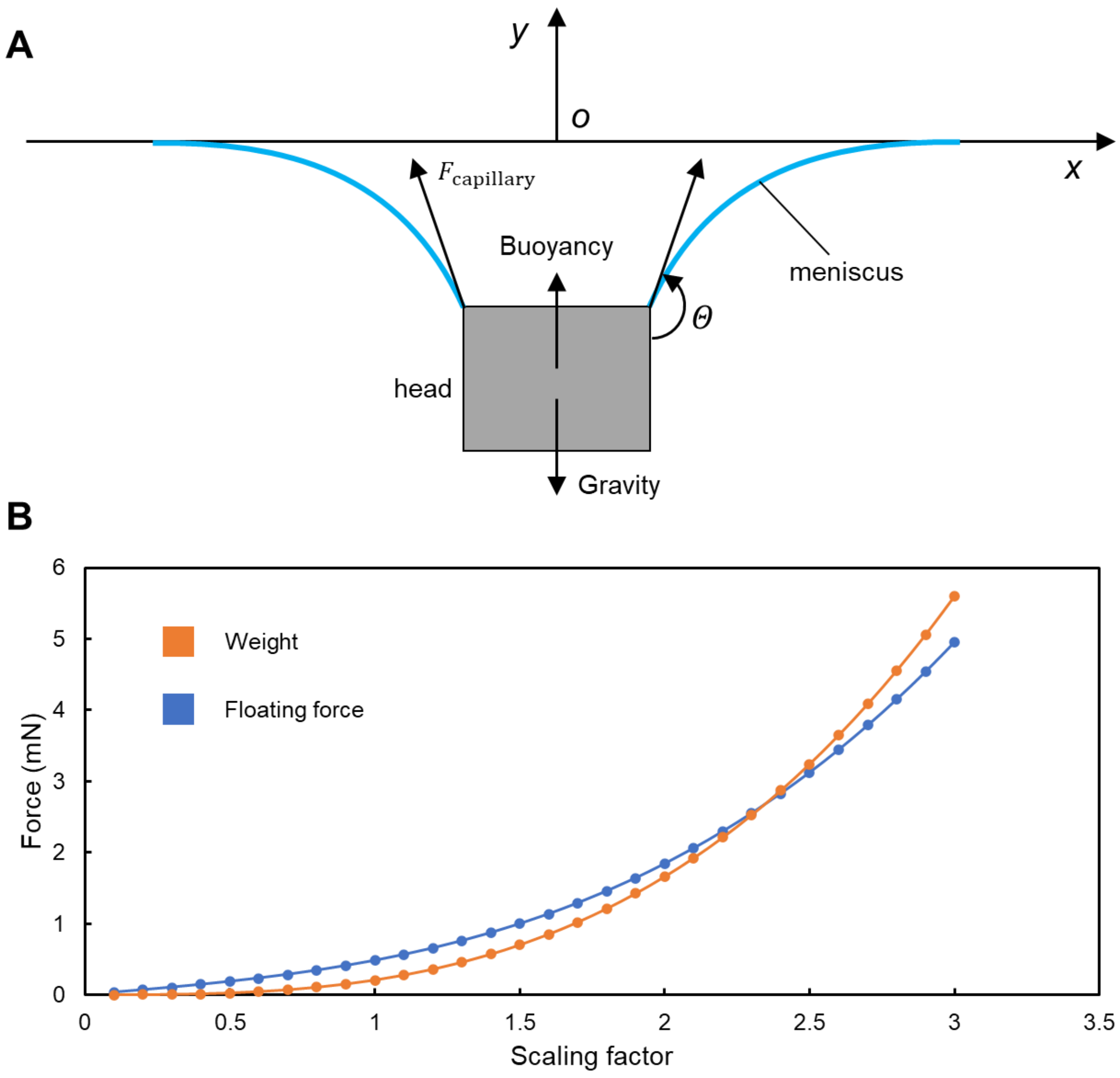

Fig. S4 Calculation of the buoyant force. (A) The force analysis of the floating object. The buoyant force is composed of the buoyancy, as per Archimedes' principle, and the normal component of the surface tension force. (B) The variation of the weight of the swimmer's head and maximum floating force the water surface can provide. The calculation assumes the swimmer's head is at the static condition.

Movie S1 (separate file).

Characterization of flow field on water surface. The swimmer has the body bending stiffness of 1.83 × 10^{-10} Nm^2 and the body length of 10 mm. The pitch angle amplitude is 26° and the actuation frequency is 50 Hz.

Movie S2 (separate file).

Influence of changing pitch angle amplitude (θ_p) and frequency (f) on body's waveform and propulsion speed.

Movie S3 (separate file).

Influence of changing body's bending stiffness (EI) and frequency (f) on body's waveform and propulsion speed.

Movie S4 (separate file).

Influence of changing body's length (L) and frequency (f) on body's waveform and propulsion speed.
